# Interventions to optimize medication use in nursing homes: a narrative review

**DOI:** 10.1007/s41999-021-00477-5

**Published:** 2021-03-09

**Authors:** Anne Spinewine, Perrine Evrard, Carmel Hughes

**Affiliations:** 1 Clinical Pharmacy Research Group, Louvain Drug Research Institute, Université Catholique de Louvain, Avenue Mounier 72/B1.72.02, Woluwe-Saint-Lambert, 1200 Brussels, Belgium; 2 Pharmacy Department, CHU UCL Namur, Université Catholique de Louvain, Yvoir, Belgium; 3 School of Pharmacy, Queen’s University Belfast, Belfast, UK

**Keywords:** Nursing homes, Older adults, Medication optimization, Potentially inappropriate prescriptions, Interventions, Implementation

## Abstract

**Purpose:**

Polypharmacy, medication errors and adverse drug events are frequent among nursing home residents. Errors can occur at any step of the medication use process. We aimed to review interventions aiming at optimization of any step of medication use in nursing homes.

**Methods:**

We narratively reviewed quantitative as well as qualitative studies, observational and experimental studies that described interventions, their effects as well as barriers and enablers to implementation. We prioritized recent studies with relevant findings for the European setting.

**Results:**

Many interventions led to improvements in medication use. However, because of outcome heterogeneity, comparison between interventions was difficult. Prescribing was the most studied aspect of medication use. At the micro-level, medication review, multidisciplinary work, and more recently, patient-centered care components dominated. At the macro-level, guidelines and legislation, mainly for specific medication classes (e.g., antipsychotics) were employed. Utilization of technology also helped improve medication administration. Several barriers and enablers were reported, at individual, organizational, and system levels.

**Conclusion:**

Overall, existing interventions are effective in optimizing medication use. However there is a need for further European well-designed and large-scale evaluations of under-researched intervention components (e.g., health information technology, patient-centered approaches), specific medication classes (e.g., antithrombotic agents), and interventions targeting medication use aspects other than prescribing (e.g., monitoring). Further development and uptake of core outcome sets is required. Finally, qualitative studies on barriers and enablers for intervention implementation would enable theory-driven intervention design.

## Key summary points

**Aim:**

This review aimed to identify, describe and discuss different interventions targeting medication use optimization in nursing homes and to identify research gaps.

**Finding:**

Prescription of the whole medication regimen or of specific medication classes was the most studied aspect. Medication review and multidisciplinary approaches appeared to be effective strategies in reducing appropriate use, but further large-scale randomized trials are needed.

**Messages:**

Efforts to optimize medication use among nursing home residents are still needed and should focus on less evaluated intervention components, specific medication classes and medication use aspects not related to prescribing.

## Introduction

Medication use among nursing home residents (NHRs) is very common. Indeed, in nursing homes (NHs), polypharmacy is highly prevalent, with 91%, 74% and 65% of NHRs taking more than five, nine and 10 medications, respectively [1]. These rates of polypharmacy are higher than what has been reported in home-dwelling older adults (27.0–59.0% taking 5 or more medications [1]). Factors associated with polypharmacy among NHRs include age, cognitive status, number of prescribers, dependency and length of stay in the NH [1].

Polypharmacy, together with other factors such as altered pharmacokinetics and pharmacodynamics, and complexity of the medication use process, makes the safe use of medications for NHRs highly challenging [2]. Reported rates of adverse drug events (ADEs) in NHs range from 1.89 to 10.8 per 100 resident-months [3]. Medication errors (MEs) are common, involving 16–27% of NHRs in studies evaluating all types of MEs and 13–31% of NHRs in studies evaluating MEs occurring at transfer from and to other settings of care [4]. MEs can occur at any step of the medication use process. These steps include: prescribing, purchase and ordering, delivery, storage, preparation and administration, monitoring and medication reconciliation at transfer [5]. The minimum practices that are required to deliver high-quality care at each step have been identified and constitute opportunities for evaluation of performance [5]. The literature suggests that the majority of errors occur at the prescribing, monitoring, administration, and medication reconciliation steps [4]. In a recent review, five categories of factors related to the work system were found to affect medication safety in NHs: persons (resident and staff, e.g., number of medications, staff medication knowledge), organization (e.g., inter-professional collaboration, staff/resident ratio), tools and technology (e.g., bar-code medication system), tasks (e.g., workload and time pressure), and environment (e.g., staff interruption) [3]. It is expected that interventions to optimize medication use in NHs would address these steps and factors as priorities.

The prescribing component is an important aspect of medication optimization, as prevalence of potentially inappropriate prescriptions (PIPs) is high, and as PIP and polypharmacy have been associated with adverse outcomes such as lower quality of life, hospitalizations, falls, and frailty [1, 6–8]. PIPs encompass underprescribing (failure to prescribe a needed drug), overprescribing (prescribing more drugs than needed) and misprescribing (incorrect prescribing of a needed drug) [2]. The estimated prevalence of PIPs among NHRs is 43.2% [9]. This prevalence tends to rise over time and the situation is more concerning in Europe, with higher reported point prevalence (49.0%) than these reported in North America (26.8%) or other countries (29.8%) [9]. Several factors were found to be associated with PIPs such as total number of medications taken, age, location of the NH (including country, urban versus rural), dementia and comorbidity burden [9, 10]. The most commonly reported inappropriate medications include psychotropic drugs, medications with anticholinergic properties, antimicrobials, nonsteroidal anti-inflammatory drugs and proton-pump inhibitors [9, 11, 12].

Interventions to optimize medication use can be implemented at different levels of the health care system. Throughout the literature there is inconsistency in the number and definitions of these levels [13]. For this review, we distinguish between two levels. First, the micro-level refers to interventions implemented at the NH level and directed at NHRs, health care providers (HCPs) and organization of the NH itself. Second, the macro-level (also called system-level) encompasses strategies that are external to NHs but impact on their practice. These are typically but not exclusively defined at a national or regional level.

The main objective of this review is to identify, describe and discuss interventions aimed at optimization of any step of medication use in NH, in terms of content, effects, as well as barriers and enablers to their implementation. As a second objective, we aimed to identify perspectives for the future at the research and practice levels.

## Method

This review was conducted using a narrative process. We focused on interventions targeting the medication use of residents living in NHs. Relevant references were identified and selected from a search in PubMed, the authors’ existing knowledge of literature, and recent publications in geriatrics journals. Finally, we retrieved additional studies by hand-searching reference lists of identified articles. Searching additional databases (e.g., Embase, CINAHL) would have been valuable and relevant in the context of a systematic review, but this was beyond the scope of the present work.

**Fig. 1:**
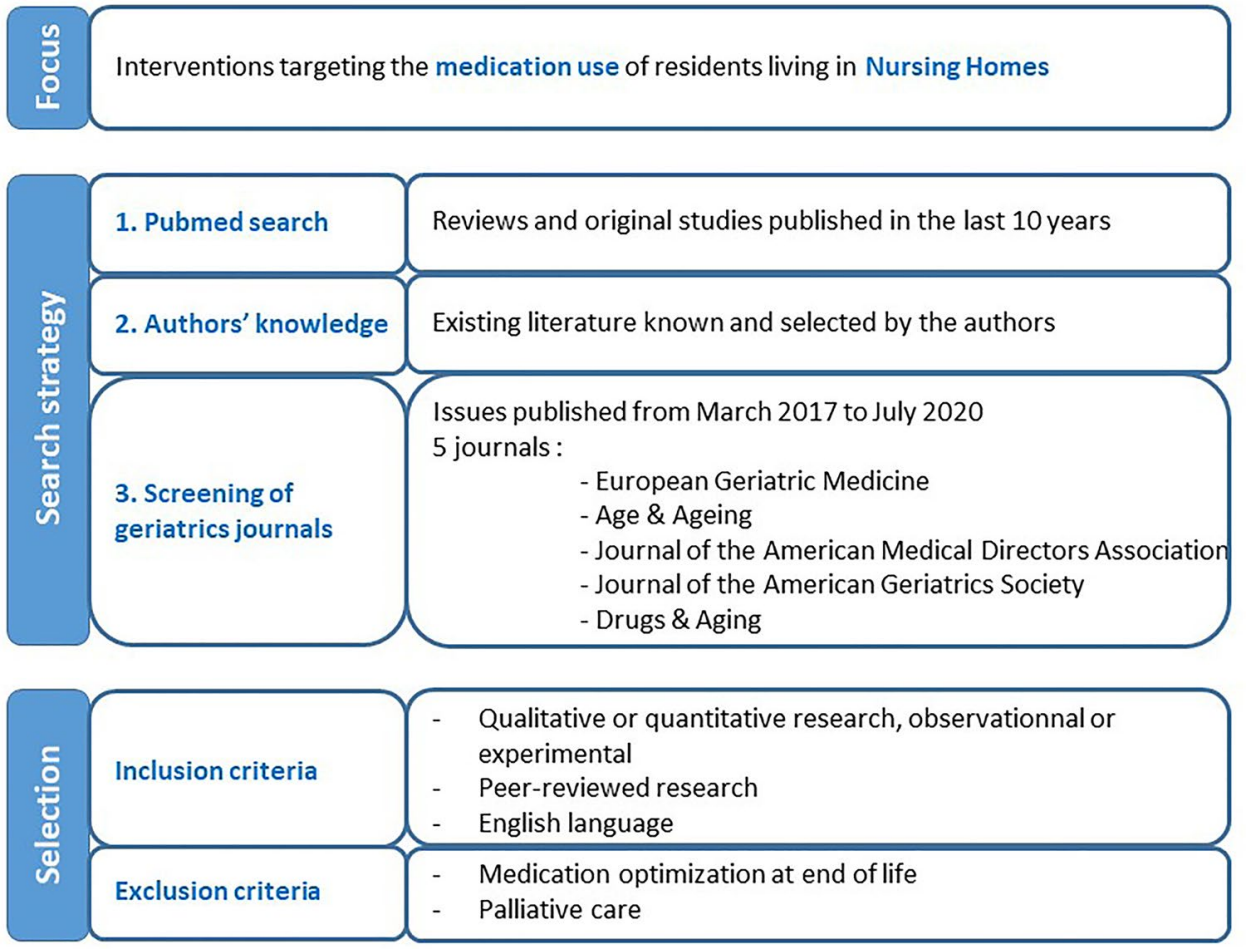
Search strategy and papers’ selection process

We selected quantitative as well as qualitative studies, observational and experimental studies that described interventions, their effect as well as barriers and enablers. We only included peer-reviewed research published in English. Given the large volume of literature, we prioritized results from the most recent (systematic) reviews and original studies published after these reviews were completed. We did not restrict the country where research took place, but gave preference to studies conducted in Europe or with relevant data or messages for European settings, as judged by the research team. We did not include papers focusing on medication optimization at end of life or during palliative care which was considered beyond the scope of this review. The search strategy and papers’ selection process are presented in Fig. 1.

Because an important part of the literature focuses on the prescribing component, we first review this aspect, followed by approaches to improve other aspects of medication use. In the section on prescribing, we review separately the approaches concerned with optimizing the whole medication regimen and those concentrating on specific drugs or classes, because the approaches, their effect, as well as barriers and enablers may differ, and hence, merit separate consideration.

### Interventions to optimize prescribing for the whole medication regimen

Three recent systematic reviews (SRs) evaluated the effect of micro-level interventions—largely based on medication review (MR)—to optimize prescribing in the NH setting and reported positive results on quality of prescribing [14–16]. A Cochrane SR highlighted four different approaches for optimization: MR, multidisciplinary case-conferencing, education for HCPs, and use of clinical decision support system (CDSS) [14]. These were used either alone, or in combination. Overall, the interventions led to identification and resolution of drug-related problems, but there was no consistent effect on resident-related outcomes [14]. In a second SR focusing on MR and including experimental and observational study designs, interventions were associated with a reduction in prescribed medications, inappropriate medications and adverse outcomes (including deaths and hospitalizations) [15]. However, high-quality cluster-randomized controlled trials evaluating CDSS effects or evaluating the impact of multidisciplinary interventions on well-defined important resident-related outcomes were lacking [14, 15]. In terms of deprescribing, a SR of specific interventions reported a reduction of 59% of NHRs receiving at least one PIP [16]. Only interventions including a MR were associated with a reduction in all-cause mortality and number of fallers [16].

Five trials performed in European Union (EU) countries NHs were published after these SRs and are summarized in Table 1 [11, 17–20]. These were all multicenter studies—three were cluster-randomized controlled trials—and involved multidisciplinary interventions mainly consisting of education of HCPs and MR. None involved a CDSS component. Participation of NHR was one component of the intervention in two studies. The study by Wouters et al. involved NHRs through a questionnaire on their preferences and experiences as a step of MR [18]. In the COSMOS study, NHRs were asked about their interest in participating in different activities [20]. Overall, results from these five trials were consistent with those of previous SRs, with positive effects on polypharmacy and PIPs—although the measures used to define PIPs varied widely across studies, and none of the tools used were specific to the NH setting. Clinical and humanistic outcomes were inconsistently evaluated (Table 2). Two trials reported no effect of the intervention on clinical outcomes and/or quality of life [11, 18]. In the COSMOS study, an initial decline in quality-of-life was found in the intervention group—initial NHR unhappiness with the MR is one of the possible explanations raised by the authors—but this decrease reversed significantly during follow-up [20].

**Table 1:** Trials conducted in European nursing homes in the last 5 years and reporting the effect of an intervention on prescribing the whole medication regimen (chronological order)

Study name, author, year	Setting	Participants no.; average age	Study design	Study duration	Intervention	Stakeholders involved in intervention	Main measures	Results of the intervention
Fog, 2017 [17]	41 NHs, Norway	2465; 85.9	BAS	28 mo	Training sessions One MR per NHR, conducted by the NH physician and a nurse, in collaboration with external pharmacist	GP, nurses, pharmacist	DRP identified (by START/STOPP and NORGEP criteria) during MR, and related interventions	6158 DRPs identified (2.6/NHR) 6283 interventions to resolve DRP
							Medication changes	Reduction of total number of drugs used by NHR by 9.3% (*p* < 0.01)
DIM-NHR: Wouters, 2017 [18] Wouters, 2019 [21]	59 NH wards, The Netherlands	426; 83.5	cRCT	4 mo	Brief training on MR MR incorporating NHRs’ experiences and preferences (multidisciplinary multistep medication review)	Elder Care Physician, Pharmacist	Successful discontinuation of at least one inappropriate drug at 4 mo [18]	Discontinuation for 39.1% of NHRs in intervention group, versus 29.5% in control group [ARR (95% CI) = 1.37 (1.02; 1.75)]
							Clinical outcomes [18]	No change in clinical outcomes
							Quality of life [18]	No change
							Barriers and facilitators of conducting MR [21]	4 themes of barriers and facilitators: Assessing the patient perspective Level of comprehensiveness of MR Inclinations of healthcare providers Inter-professional collaboration, alliances
IQUARE: Cool, 2018 [19]	159 NHs, France	629; 87.0	cCT	18 mo	Audit and feedback on quality indicators for both IG and CG Two half-day meetings with geriatrician and NH staff to discuss results and decide strategies for improvement	GP, nurses, hospital geriatrician, coordinating physician	PIP, as any: (i) unfavorable benefit-to-risk ratio (ii) questionable efficacy (iii) absolute contraindication (iv) significant drug–drug interaction	The intervention significantly decreased PIPs [65.2% in IG, 69.9% in CG, OR (95% CI) = 0.63 (0.40; 0.99)]
COSMOS: Husebø, 2019 [20] Gulla, 2019 [22]	33 NHs, Norway	723; 86.7	cRCT	9 mo	Education and training of NH staff**:** 2 days’ seminar combined with repeated lunch meetings for 4 mo, focusing on MR, pain assessment and communication with NHRs Interdisciplinary MR (every 6mo), with collegial monitoring Organization of activities, based on NHRs interests	GP, management, nurses	Change in QoL at 4 months, measured by 3 indicators [20]	Deterioration in QoL measures at 4mo, then improvement from 4 to 9 mo
							Secondary outcomes: ADL, total medication, staff distress, CGIC [20]	Improvements in all secondary outcomes
							Process evaluation [22]	92% of NHR in IG underwent a MR Improvement of communication between stakeholders Reported barriers: lack of time, low engagement of stakeholders, ethical dilemmas
COME-ON: Strauven, 2019, [11], Anrys, 2019 [23]	54 NHs, Belgium	1804; 87.5	cRCT	15 mo	Training: e-learning (4 modules of 1 hour each) and face-to-face workshops (2 h each) Local interdisciplinary meetings, where use of a medication class was discussed Three interdisciplinary case conference per NHR, with MR	Coordinating physician, GP, nurses, pharmacist	Resolution of at least one PIP present at baseline, without new PIP (identified by START/STOPP and AGS Beers criteria) [11]	Significant positive effect of the intervention [OR (95% CI) = 1.479 (1.062; 2.059)]
							Secondary outcomes: clinical outcomes, medication use [11]	No significant difference between IG and CG for most clinical outcomes and for median number of medications
							Process evaluation: barriers and enablers at intervention, professional, organization and external context levels [23]	Good rate of implementation and participants’ satisfaction despite variations between NHs and stakeholders Various barriers and enablers identified at intervention, professional, organization and external context levels

*ADL* activities of daily living, *ARR* adjusted relative risk, *BAS* before–after study, *CG* control group, *CGIC* clinical global impressions of change, *CI* confidence interval, *CT* controlled trial, DRP drug-related problems, *GP* general practitioner, IG intervention group, MR medication review, *mo* months, *N* nurse, *NH* nursing home, *NHR* nursing home resident, *OR* odds ratio, *PIP* potentially inappropriate prescription, *QoL* quality of life, *RCT* randomized controlled trial, *cRCT* cluster-RCT, *START/STOPP* screening tool to alert doctors to right treatment/screening tool of older persons’ prescriptions

**Table 2:** Outcomes and process measures reported in trials conducted in European nursing homes in the last 5 years

Study	Intermediate outcomes		Clinical outcomes			Humanistic outcome	Process measures	
	Medication use (general medication or specific)	PIP	Healthcare use	Mortality	Others	Quality of life	Implementation rate	Barriers and enablers
Fog, 2017 [17]	Number of medications, Medication change	Categories of drug-related problems						
DIM-NHR: Wouters, 2017 [18] Wouters, 2019 [21]		DBI PIP discontinuation	Visit to outpatient clinic Visits by HCPs		Falls Cognitive function (SIB-S, MMSE)	EQ-5D-3L DQI	X	X
IQUARE: Cool, 2018 [19]		PIP as defined by the authors						
COSMOS: Husebø, 2019 [20] Gulla, 2019 [22]	Number of medications Use of specific medication classes				CGIC Neuropsychiatric symptoms ADL	QUALIDEM QUALID EQ-VAS	X	X
COME-ON: Strauven, 2019, [11], Anrys, 2019 [23]	Number of medications Use of specific medication classes	START/STOPP criteria AGS Beers criteria	Hospital admissions Visits to ED Visits to GO or specialist physicians	X			X	X
PROPER II, Van der Spek, 2018 [26]		APID index						
EPCentCare, Richter, 2019 [27]	Antipsychotic users		Fall-related		Falls Agitated behavior Restraint use	QoL-AD	X	X
Weeks, 2018 [28]	Change in psychotropics dose Medication discontinuation			X	Falls Restraint use			
Wauters, 2019 [29]	Long-term psychotropics Concomitant psychotropics							
Plüss-Suard, 2020 [30]	Antibacterial use							

*ADL* activities of daily living, *AGS* American Geriatrics Society, *APID* appropriate psychotropic drug use in dementia index, *CGIC* Clinical Global Impression of Change, DBI Drug Burden Index, *DQI* dementia quality of life instrument, *ED* emergency department, *EQ-5D-3L* EQ-VAS European quality of life visual analog scale, *HCPs* health care providers, *QoL-AD* quality of life in Alzheimer’s disease scale, *QUALID* quality of life late-stage dementia scale, *QUALIDEM* quality of life dementia scale, *MMSE* mini-mental state examination, *PIP* potentially inappropriate prescription, *SIB-S* severe impairment battery-short, *START/STOPP* screening tool to alert doctors to right treatment/screening tool of older persons’ prescriptions. ‘X’ without further detail means that the outcome was reported, but no details on measurement were given.

Beyond the evaluation of the effect of interventions, a clear understanding of the enablers and barriers to implementation and success is crucial for the development of future interventions. It is encouraging to see that three of the trials presented in Table 1 addressed this question, mainly through questionnaires and interviews of HCPs [21–23]. Wouters et al. also interviewed NHRs [21]. Overall, the interdisciplinary approaches were recognized as key elements for the success of interventions, despite organizational and time constraints. The attitude, role and competency of HCPs (physicians, pharmacists and nurses) were identified both as barriers and enablers. The need for funding MRs at the macro-level was also reported. Assessing the patient perspective was reported to be a delicate balance between the value and the barriers to a proper assessment of the patient perspective. Other qualitative studies assessed the specific barriers and enablers of deprescribing in the NH setting [24, 25]. While many were similar to what was reported for intervention implementation, HCPs’ concerns about deprescribing and perceived reluctance of NHRs to change were more specific to deprescribing interventions. This highlights the need for deprescribing guidance and shared decision-making [24, 25].

### Interventions to optimize prescribing for specific drug classes

In the section below, we focus on three medication classes for which inappropriate use is highly prevalent and is a threat to patient safety. For each of these, we first briefly describe data on their (inappropriate) use, then review the evidence on approaches for optimization, as well as barriers and enablers for improvement. Table 3 describes five recent studies conducted in NHs in Europe, four on psychotropic drugs and one on anti-infective drugs. We found no recent EU study focusing on DAP.

**Table 3:** Trials conducted in European nursing homes in the last 5 years and reporting the effect of an intervention on prescribing a specific medication class (chronological order)

Study name, author, year	Setting	Participants no.; average age	Study design	Study duration	Intervention	Stakeholders involved in intervention	Main measures	Results of the intervention
PROPER II, Van der Spek, 2018 [26]	12 NHs with dementia special care units, Netherlands	380 dementia NHRs; 83.6	cRCT	18 mo	Training on MR, efficacy and side effects of psychotropic drugs Interdisciplinary MR at 0-, 6-, and 12 months Evaluation phase prior to 6- and 12 months MR	Elder care physician, nurses, pharmacist	Appropriateness of psychotropic drug prescriptions, assessed with the APID index	Greater improvement of the APID index sum score over time in the intervention group compared to control (− 5.28, *p* = 0.005)
							Secondary outcomes: appropriateness of indication, evaluation and therapy duration; assessed with APID index subscores	Significant improvement in evaluation and therapy duration subscores
EPCentCare, Richter, 2019 [27]	37 NHs, Germany	1153 dementia and non-dementia NHRs; 84.1	cRCT	12 mo	MR conducted at baseline, 3, 6 and 9 mo by experienced physicians specialized in psychotropic drug treatment for older people, in both CG in IG 2 h training for physicians in CG and IG Training of IG NH staff: 2-day workshop on person-centered care, and continuous in-house support	Physicians, NH staff	Proportion of NHRs with at least one antipsychotic prescription	Proportion changed from 44.6% to 44.8% in the intervention group and from 39.8% to 33.3% in control group Difference in prevalence between intervention and control groups: 11.4% [(95%CI) = (0.9–21.9); OR (95%CI) = 1.621 (1.038; 2.532)]
							Secondary outcomes: quality of life, agitated behavior, falls and fall-related medical attention	No significant differences between IG and CG
							Process evaluation	Variation of degree of implementation across intervention components and NHs (continuing medical educations was attended by very few physicians)
							Health economics evaluation	Intervention cost added up to 52.518 Euro
Weeks, 2018 [28]	45 NHs, Spain	1653 dementia NHRs; 86.5	Retrospective, PSM, controlled	1 mo	Comparison of 3 interventions: Interdisciplinary MR Use of STOPP/START criteria Use of a patient decision aid	Not reported	Change in specific milligram-equivalent daily dose for 3 psychotropic drug classes (antipsychotic, antidepressant, anxiolytic)	Significant reduction in daily doses for all 3 classes and for every intervention (from a 11.9% reduction for antidepressants with MR to a 39.5% reduction for anxiolytics with the use of STOPP/START criteria) Most effective intervention: STOPP/ START criteria
							Patient falls and restraint use at 2 weeks	No higher rates of patient falls or physical restraints
Wauters, 2019 [29]	5 NHs, Belgium	677 dementia and non-dementia NHRs; 85.6	BAS	12 mo	Training: educational session for GPs and nurses on psychotropic drugs (evidence-based practice, reduction in use, non-pharmacological alternatives) MR Transition towards person-centered care	Coordinating physicians, GPs, nurses, pharmacist	Long-term (> 3 mo) psychotropic drug use	Significant decrease from 62.0% to 52.9% (*p* < 0.001)
							Concomitant psychotropic drug use	Significant decrease from 31.5% to 24.0% (*p* = 0.001)
Plüss-Suard, 2020 [30]	23 NHs, Switzerland	All NHRs from the canton of Vaud during study period	Longitudinal Quality Improvement study	6 years	Publication of local guidelines on empirical antibacterial therapy Interdisciplinary quality circles	Coordinating physicians, nurses, pharmacist	Antibacterial use, expressed as DDD per 1000 beds per day	Decrease from 45.6 to 35.5 DDD per 1000 beds per day (− 22%, *p* < 0.01)

*APID* appropriate psychotropic drug use in dementia, *ARR* adjusted relative risk, *BAS* before–after study, *CG* control group, *CGIC* clinical global impressions of change, *CI* confidence interval, *CT* controlled trial, *DDD* defined daily dose, *DRP* drug-related problems, *GP* general practitioner, *IG* intervention group, *MR* medication review, mo months, *N* nurse, *NH* nursing Home, *NHR* nursing home resident, *OR* odds ratio, *P* pharmacist, *PIP* potentially inappropriate prescription, *PSM* propensity score-matched, *QoL* quality of life, *RCT* randomized controlled trial, *cRCT* cluster-RCT, *START/STOPP* screening tool to alert doctors to right treatment/screening tool of older persons’ prescriptions

#### Psychotropic drugs

Psychotropic drugs are used extensively in NHs, with wide variation in rates of prescribing between countries. In NHs in Western European countries, antipsychotic use ranges from 12 to 59% of NHRs and antidepressant use is even higher, from 19 to 68% [31]. The use of benzodiazepine receptor agonists (BZRA, namely benzodiazepines and Z-drugs) ranges from 14.6% (Canada, [32]) to 54.4% (France, [33]). Concomitant use of several psychotropic drugs is also high with 31.5% of NHRs taking two or more such medications [29].

Beyond this high prevalence of use, frequent inappropriate use is a concern. Indeed, psychotropic drugs are often the most commonly reported inappropriate medications among NHRs [9, 11, 34]. The inappropriate (and off-label) use of antipsychotics for behavioral and psychological symptoms of dementia has received the most attention. This has led to national and international calls and programs for deprescribing of antipsychotics in NHs. Even though the appropriateness of antidepressants and BZRA use has been less widely studied, recent data suggest that these medicines should also give rise to concern. In a study with 2651 French NHRs receiving an antidepressant, PIP (with regard to indication, drug class, duplication and monitoring) was found in 38.4% of NHRs [35]. In a Belgian study with 418 NHRs taking a BZRA, 98% of NHRs received the BZRA for more than 4 weeks, and drug–disease and drug–drug interactions were found in two-thirds of users overall [36]. In both studies, dementia was associated with less PIP.

Data on the factors associated with (inappropriate) psychotropic drug use suggest that approaches for improvement can be considered both at the macro- and micro-levels. A recent SR found that organizational capacity, individual professional capacity, attitudes, communication and collaboration and regulation or guidelines influenced antipsychotic prescribing [37]. Similarly, factors associated with psychotropic drugs use included: staffing level or education, teamwork and communication between both on-site and visiting staff, and managerial expectations [13].

At the macro-level, a recent scoping review including 36 studies (of which only three were performed in Europe) found that mandatory strategies such as legislation (e.g., change in reimbursement, initiation of public reporting of antipsychotic use) had greater evidence of impact on drug utilization than non-mandatory macro-level strategies such as guidelines and recommendations [13]. The OBRA-87 legislation in the US led to the greatest reduction in psychotropic drug use. However, inappropriate use remains a significant issue and few studies have examined both sustainability of system-level strategies and cost-related outcomes [13].

At the micro-level, a recent narrative review of approaches for deprescribing psychotropic medications in NHRs with dementia reported that interventions should have more than one component, include multidisciplinary teams and HCPs’ training, and be person-centered [38]. The same intervention components were highlighted in a SR of factors influencing antipsychotic use among dementia NHRs [37] and in a review of interventions targeting BZRA deprescribing [39]. In Europe, a few interventions were recently evaluated, with encouraging results (Table 3 [26–29]). Similar to approaches targeting the whole medication regimen, training of HCPs and MR were important components of evaluated strategies. However, some more specific strategies were also tested. Patient-centered interventions were implemented in three studies. In Belgium, a quality improvement study with transition to person-centered care (e.g., through the implementation of meaningful activities for NHRs) showed a reduction in both long-term use and concomitant use of psychotropics [29]. In a Spanish study with NHRs with dementia, application of STOPP/START criteria and use of decision aids for NHRs had positive and similar effects on reducing daily dosages of psychotropic drugs, even though decision aids were less often used than STOPP/START [28]. Richter et al. investigated a person-centered care approach, which had been successfully evaluated in NHs in the UK, and adapted it to the German context. However, the program did not lead to a reduction in antipsychotic prescriptions. Reasons for differences between the UK and Germany were unclear, but the culture of care as reflected in the attitudes and beliefs of nursing staff and a lack of cooperation with physicians may have accounted for the findings [27].

#### Drugs with anticholinergic properties (DAP)

DAPs are associated with a wide range of peripheral and central adverse effects (e.g., delirium, fall, urinary retention), and there have been numerous calls to reduce their use [40]. A recent population-based study among NHRs with depression even found that clinically significant anticholinergic use was associated with a 31% increase in risk of death [41]. Despite such concerns, DAP are highly prevalent among NHRs. In a study conducted in Helsinki, in 2011, 85% of NHRs were taking at least one DAP [42]. Positive findings were reported in a study evaluating temporal trends from 2003 to 2017 (the anticholinergic burden decreased, and participants with dementia had a lower anticholinergic burden), but DAP use—especially antipsychotics and antidepressants—remained high [43]. This calls for action toward DAP use in NH.

A SR reported that (micro-level) interventions aiming at reducing anticholinergic burden in older adults (≥ 65) in different settings often reduced anticholinergic burden [40]. Pharmacists delivered the intervention in the majority of studies, and authors concluded that these HCPs may be well placed to implement a DAP reduction intervention [40]. Among the eight studies included, only one was conducted in NHs, in Norway. The intervention consisted of a pharmacist-initiated reduction of anticholinergic drug scale score after multidisciplinary MR. Anticholinergic drug scale scores were significantly reduced in the intervention group and remained unchanged in the control group. However, no improvement in NHRs’ cognitive function at 8 weeks was observed [44]. In another recent study conducted in New Zealand NHs, pharmacists performed deprescribing recommendations for both anticholinergic and sedative drugs. This showed that deprescribing was feasible, with 72% of recommendations implemented by physicians, without deterioration in quality of life, and with an improvement in depression and frailty scores [45]. No macro-level approaches specifically targeting DAP use were found.

These data are encouraging but remain very limited, which calls for further well-conducted, large-scale, controlled studies. The variety and heterogeneity of tools to measure and quantify anticholinergic burden remains an issue, as there is no consensus as to which of the tools is most useful in research or clinical settings [42].

#### Anti-infective drugs

Antimicrobials are commonly prescribed in NHs and their use is associated with antimicrobial resistance and *Clostridium difficile* infections. The 2016–2017 point prevalence survey performed in NHs in 24 EU countries found a crude prevalence of NHRs receiving at least one antimicrobial agent of 4.9%, with large variations across and between countries (from 0.7% in Lithuania to 10.5% in Spain and Denmark) [46]. Prophylaxis for urinary-tract infection was a frequent—and potentially inappropriate [47]—indication for antimicrobial use (representing almost one third of prescriptions) and did not significantly decline following previous surveys [46]. Inappropriate prophylactic use of antimicrobials was therefore recommended as a specific target for future interventions. Appropriate prescribing of antimicrobials in NH is challenging and influenced by several factors, such as variations in knowledge and practice among HCPs, social factors, antimicrobial resistance and the specific context of NH care (including restricted access to doctors and diagnostic tests) [12].

Antibiotic stewardship programs (ASPs) are coordinated interventions promoting the appropriate use of antibiotics to improve patients’ outcomes and reduce microbial resistance [48], which can be implemented at both the macro- and micro-levels. At the macro-level, ASPs have been mandated in American NHs since November 2017. In Europe, data on ASP indicate that there has been no increase in ASP implementation over time, and improvements in antimicrobial stewardship are urgently needed in EU NHs [46].

Recent SRs on ASP in the NH setting reported that the most commonly implemented strategies were educational materials, educational meetings, and guideline implementation, combined in multifaceted interventions [49]. Results suggested an effect on intermediate health outcomes, such as antibiotic consumption or adherence to antibiotic guidelines. However, an effect on key health outcomes such as mortality rates, hospitalizations, or *Clostridium difficile* infection rates was not demonstrated [48–50]. Moreover, the specific benefit of intervention components is unclear. In Switzerland, ASP activities including local multidisciplinary networks (micro-level strategy) and guidelines publication (macro-level strategy) led to a 22% reduction in antibacterial use over a 6-year period (Table 2) [30]. A recent paper described the ASP implementation experience in four European countries (Norway, The Netherlands, Poland and Sweden) where various regional or national ASP initiatives have recently been introduced [51]. The ASP components included national surveillance systems, NH-specific prescribing guidelines and national networks of healthcare institutions. No data were provided to document the effect of these initiatives on antimicrobials consumption. Future ASP implementation will need to account of enablers (e.g., the presence of study leaders, skills training for doctors and nurses, and good inter-professional communication) and barriers (e.g., pressures from residents and families, NH staff’s knowledge and belief) in order to be successful, in addition to outcome data [12, 52].

### Interventions to optimize medication reconciliation at transfer

The transition of NHRs from one setting to another increases the risk for MEs. Indeed, preventable ADEs at transition points account for 46–56% of all MEs [53] and MEs have been identified as a major source of morbidity and mortality in transitional care [54]. A possible explanation is poor communication between settings, potentially leading to prescribing errors. When questioned on ways to improve quality and safety of care transfer, NH and emergency department staff raised several strategies, including the use of a standardized transfer form, a checklist and verbal communication between settings [55].

In practice, some of these interventions have been studied at micro-level. Results from a SR on interventions to improve transitional care between NH and hospitals show that the development of a standardized unique transfer document may assist with the communication of medication lists, and that pharmacist-led review of medication lists may help identify omitted or indicated medications on transfer [54]. This is supported by results from another SR evaluating medication reconciliation interventions during NHRs’ transfer from and to the NH [53]. In most studies, a clinical pharmacist performing MR was part of the intervention. All interventions led to outcome improvement, but no study showed strong evidence in reducing medication discrepancies [53].

Existing data also suggest that HCPs believe that initiatives should be taken at the macro-level, to standardize processes during transitions. National guidance and toolkits relative to medication reconciliation in the NH setting exist in some countries such as Canada [56], but to the best of our knowledge, the impact of these initiatives on quality and safety of medication use in NHs has not been evaluated.

### Interventions to optimize the preparation and administration

The preparation and administration of prescribed drugs often falls to nurses (and sometimes pharmacists for the preparation stage)—and not to NHRs themselves. Medication administration errors (MAEs) encompass different types of errors such as wrong-time errors, wrong-dose errors, omitted doses, wrong-patient errors. As an example, 27% of calls to the Quebec Poison Center for patients aged over 65 resulted from drug administration to the wrong NHR [57]. The medication administration process is prone to interruptions, and this may increase the risk of MAE. It has been reported that nurses are interrupted at a rate ranging from 0.4 to 14 times an hour [58]. Swallowing difficulties may also trigger MAEs. Indeed, it is common for nurses to modify medication dosage forms through crushing tablets or opening capsules, in order to administer a medication to NHRs with swallowing difficulties [59]. Nurses reported that this practice is challenging and would need appropriate guidelines and training [59].

To reduce the risk of MAEs and resulting harms, different approaches have been taken, and the main focus has been the implementation of technological solutions, such as electronic medication administration record (eMAR) and bar-code medication administration [58, 60–62]. These technologies might be time-saving, decrease the probability of MAEs such as omitted doses and increase nurse satisfaction [61, 62]. However, a SR on eMAR in long-term care facilities reported that eMAR implementation is low, partly because of cost barriers, and there is a lack of rigorously designed research to inform administrators and clinicians about the effect of eMARs and bar-code medication administration on MEs [60]. The use of multi-compartment compliance aids is another possible approach to reduce preparation and administration errors. A recent study in London reported that MAE rate was higher with original medication packaging than with multi-compartment compliance aids (risk ratio = 3.9, 95% CI 2.4–6.1) [63]. Limitations to their use included reduced staff alertness during administration and difficulties in identifying medication [63].

## Discussion

This review has highlighted that many interventions focusing on the key steps in medicine optimization led to improvement in medication use. However, some components have not been comprehensively evaluated or not in powerful designs such as randomized controlled trials. In much of the literature reviewed, there was an under-representation of aspects of medication use not related to prescribing (including monitoring). This is perhaps not surprising due to the predominance of the prescribing process in healthcare, but other aspects of medication use do require further consideration. Many studies that did focus on prescribing had common intervention components. At the micro-level MR, multidisciplinary work, and more recently, patient-centered care components dominated; at the macro-, guidelines and legislation, mainly for specific medication classes, e.g., antipsychotics, were employed. Improving administration was achieved through utilization of technology.

What was also apparent in the studies examined was the marked heterogeneity in outcome reporting and measurement across studies (Table 2). This makes synthesis of findings difficult and highlights the need for a more common approach across studies examining similar research questions. This may be realized through the development and use of core outcome sets (COSs). Two relevant COSs exist, for trials aimed at optimizing prescribing among NHR [64] and for trials of MR in multi-morbid older patients with polypharmacy [65]. Several outcomes of these COSs have been under-evaluated (i.e., *what* to measure), such as pain relief, all-cause mortality, falls, quality of life, hospital admissions and emergency visits to hospital. These are clearly important outcomes for this particular population and for the health systems. It is important that future trials refer to and use a COS. Furthermore, approaches to measurement of outcomes (i.e., *how* to measure) were also highly variable. PIP was measured in most studies, but a wide range of tools was used. Although many were targeted at older adults, such tools may not be appropriate for NHRs who have a higher degree of frailty. The use of tools that have been specifically developed for those who are frail [47] or living in residential care (stoppNH [66]) may be a better option.

System level (macro-level) approaches were implemented in US and Australia, but much less so in Europe. Positive effects were seen with mandatory/legislative initiatives, and it could be argued that these should be considered at the European level. However, there has been a tradition of different countries tackling approaches in nursing home care in different ways which may be a function of different cultural and political contexts [67]. Many of the concerns around prescribing of key medicines such as antipsychotics and anti-infectives are universal, and a more comprehensive, cross-country approach may be warranted.

At the micro-level, the importance of patient-centered interventions was increasingly recognized. Patient involvement or participation in the interventions was identified in two recent EU studies focusing on psychotropic drugs [28, 29], and in one of the studies to improve prescribing for the whole medication regimen [18]. However, more research on how best to involve NHRs, and NHRs with dementia in particular, is required. In some countries, patient and public involvement is increasingly expected as part of applications for research funding [68]. A recent study introduced weekly participatory action research sessions. During these, NHRs could discuss NH initiatives and suggest improvement. Results reported a positive NHR experience and an improved quality of life [69]. However, this is a challenging area as many NHRs will have varying levels of cognitive impairment, which may limit the level of their participation.

This paper focused on a number of specific medication classes which, historically, have been viewed as problematic in this population. With regard to psychotropics, a particular focus has been on reducing the use of antipsychotic drugs, but there was little exploration of any compensatory increases in the use of other sedating psychotropic drugs [70] or in the use of non-pharmacological approaches. Measurement of clinical and humanistic outcomes was limited and heterogeneous [27], therefore, a COS for interventions targeting psychotropic/antipsychotic drug use in NHs would be welcome. Indeed, this was also seen with studies focusing on DAPs, with a plethora of scales available, but little overlap to facilitate comparison. Anti-infectives have also been extensively studied in the NH environment. There has been no concerted attempt to introduce macro-level interventions focusing on ASP, which may reflect differing prescribing practices and cultures [71], but there have been efforts to begin to standardize the important outcomes for ASP interventions [72].

We selected the medication classes above because of a legacy of concern over inappropriate use. However, other medication classes also deserve specific focus, but have been ignored. Pain control is one of the outcomes of a COS of MR in older people [65]. Inappropriate prescribing of analgesics, and opioids in particular has been described in NHRs [73–75]. Second, there has been little work focusing on optimizing the use of antithrombotic agents among NHRs. This is an important research gap, as bleeding and thrombotic events are the most frequent ADEs [4]. Third, data on the deprescribing of medications used for cardiovascular prevention (e.g., statins, aspirin) and for diabetes would also be welcome, as no or very limited data are available [76–78]. Finally, the use of proton-pump inhibitors (PPIs) is highly prevalent and often inappropriate [79, 80]. While factors associated with both PPIs use and discontinuation have been described [79, 81], we found only one single-center intervention study targeting PPIs deprescribing [82]. The implementation of a deprescribing guideline was not associated with a statistically significant decrease in PPIs use [82].

Health information technology (HIT) has the potential to improve medication use in this environment, specifically to reduce the occurrence of medication errors. HIT includes systems such as eMARs, electronic medication management systems, CDSS, electronic health records [62]. Long-term care facilities have lagged behind other sectors in the adoption of HIT because of the lack of funding [62]. The eMAR system was one of the most common types of technology implemented. However, this type of technological support did not extend to supporting clinical decision-making. There was little data on the effect of CDSS in NHs, but there is ongoing research on this topic (83). Its impact in the long-term environment remains to be seen as recent trials on CDSS to optimize prescribing in primary and acute care have shown negative results on clinical outcomes [84, 85]. The relevance of alerts and usability seem to be limiting features, and these finding would be important if this technology were implemented in NHs. Other aspects of technological interventions are also lacking a strong evidence base such as the completeness and accuracy of transfer of medication information at transition moments, and the role of telemedicine.

Evidence is lacking regarding the transferability of interventions across countries and across NHs because barriers and enablers differ. Sometimes, culture and context will overwhelm any attempt to implement an approach that has worked else. However, increasingly, more attention is being paid to how interventions are developed by using recognized frameworks such as the Medical Research Council guidance on the complex interventions [86]. This systematic approach advocates for reference to existing evidence, the use of theory, modeling, pilot/feasibility testing, and implementation. There are now many more examples of interventions being developed using this approach, with a particular emphasis on theories of behavior change [87], and how barriers and enablers can be recognized [88]. A large trial evaluating the effectiveness and cost-effectiveness of a pharmacist-independent prescribing service in NHs compared to usual general practitioner-led care has been conducted in the UK and is due to report soon [89]. This trial also has an embedded process evaluation, to try to understand the mechanisms of action associated with the interventions and to explain findings in terms of fidelity to intervention performance [89]. This rigorous approach to design and evaluation enhances confidence in the conduct and findings of such studies and should be adopted by others seeking to develop and assess interventions in NHs.

## Conclusion

The NH setting and its residents have been a focus for a range of interventions targeting the spectrum of optimizing medicines use. This review has highlighted that a number of interventions are effective, but there is a need for further well-designed and large-scale evaluations of intervention components (e.g., health information technology, patient-centered approaches), specific medication classes (e.g., antithrombotic agents) which have been less commonly studied. Interventions targeting medication use aspects other than prescribing (e.g., monitoring) should also be evaluated. Building the evidence base for effective interventions would benefit from the development and uptake of COSs to allow for synthesis of findings. Finally, qualitative studies on barriers and enablers for intervention implementation would enable theory-driven intervention design. This is likely to lead to more robust and rigorous assessments of what is effective in a patient population that has unique health care needs and challenges.
